# High prevalence of penicillin-nonsusceptible Streptococcus pneumoniae at a community hospital in Oklahoma.

**DOI:** 10.3201/eid0603.000308

**Published:** 2000

**Authors:** R L Moolenaar, R Pasley-Shaw, J R Harkess, A Lee, J M Crutcher

**Affiliations:** Centers for Disease Control and Prevention, Atlanta, GA 30333, USA. rlm8@cdc.gov

## Abstract

During 1997, Oklahoma City's Hospital A reported penicillin-nonsusceptible Streptococcus pneumoniae in almost 67% of isolates. To confirm this finding, all Hospital A S. pneumoniae isolates from October 23, 1997, through February 19, 1998, were tested for antibiotic susceptibility and repeat-tested at two other hospital laboratories. Medical records of Hospital A patients with invasive S. pneumoniae infections during 1994 through 1997 were also reviewed. These data were compared with 1998 statewide sentinel hospital surveillance data for invasive S. pneumoniae. Of 48 S. pneumoniae isolates from Hospital A during October 23, 1997, through February 19, 1998, 31 (65%) were penicillin-nonsusceptible S. pneumoniae, and 23 (48%) were highly penicillin resistant. Similar prevalences were confirmed at the other hospital laboratories; however, significant interlaboratory differences were noted in the determination of third-generation cephalosporin susceptibility. During 1994 through 1997, a trend toward increasing penicillin nonsusceptibility (p <0.05) was noted among S. pneumoniae isolates from nursing home patients. During 1998, 85 (30%) of 282 invasive isolates reported to the state surveillance system were penicillin-nonsusceptible S. pneumoniae; 33 (12%) were highly resistant. The increase in resistance observed is notable; the interlaboratory discrepancies are unexplained. To respond, a vaccination program was implemented at Hospital A, and vaccination efforts were initiated at nursing homes.


### High Prevalence of Penicillin-Nonsusceptible *Streptococcus pneumoniae* at a Community Hospital in Oklahoma

**Ronald L. Moolenaar,*‡ Ronda Pasley-Shaw,† John R. Harkess,† Anthony Lee,‡ and James M. Crutcher‡**
*Centers for Disease Control and Prevention, Atlanta, Georgia, USA; †Mercy Health Center, Oklahoma City, Oklahoma, USA; and ‡Oklahoma State Department of Health, Oklahoma City, Oklahoma, USA

During 1997, Oklahoma City's Hospital A reported penicillin-nonsusceptible ***Streptococcus pneumoniae*** in almost 67% of isolates. To confirm this finding, all Hospital A ***S. pneumoniae*** isolates from October 23, 1997, through February 19, 1998, were tested for antibiotic susceptibility and repeat-tested at two other hospital laboratories. Medical records of Hospital A patients with invasive ***S. pneumoniae*** infections during 1994 through 1997 were also reviewed. These data were compared with 1998 statewide sentinel hospital surveillance data for invasive ***S. pneumoniae***. Of 48 ***S. pneumoniae*** isolates from Hospital A during October 23, 1997, through February 19, 1998, 31 (65%) were penicillin-nonsusceptible ***S. pneumoniae,*** and 23 (48%) were highly penicillin resistant. Similar prevalences were confirmed at the other hospital laboratories; however, significant interlaboratory differences were noted in the determination of third-generation cephalosporin susceptibility. During 1994 through 1997, a trend toward increasing penicillin nonsusceptibility ($p < 0.05$) was noted among ***S. pneumoniae*** isolates from nursing home patients. During 1998, 85 (30%) of 282 invasive isolates reported to the state surveillance system were penicillin-nonsusceptible ***S. pneumoniae***; 33 (12%) were highly resistant. The increase in resistance observed is notable; the interlaboratory discrepancies are unexplained. To respond, a vaccination program was implemented at Hospital A, and vaccination efforts were initiated at nursing homes.

***Streptococcus pneumoniae*** is a major cause of bacterial pneumonia and meningitis in the United States. The spread of penicillin-nonsusceptible ***S. pneumoniae*** (PNSP) has been well documented (1-8). Within a large city, resistance patterns can vary by region (5,8). Oklahoma City has historically had a relatively high but stable prevalence of PNSP among invasive isolates; penicillin nonsusceptibility was reported in 12.2% of invasive isolates during 1984 (9). From July 1989 through June 1990, 7.6% of invasive isolates from central Oklahoma were penicillin nonsusceptible, but high penicillin resistance (1.4%) was beginning to emerge (10). In late 1997, higher than expected levels of PNSP were reported in northwest Oklahoma City at Hospital A, a 392-bed community hospital, primarily providing adult care. The hospital's inpatient census and the proportion of patients receiving Medicare had not increased since 1994; however, the microbiology laboratory had recently begun using the antimicrobial gradient strip method for measuring antibiotic susceptibility to penicillin and cephalosporins. Nonsusceptibility to penicillin among pneumococcal isolates exceeded 60%; approximately half were highly penicillin resistant. We initiated an investigation to determine whether the reported prevalence was accurate and if so, to explain it, determine a

Address for correspondence: Ronald L. Moolenaar, Centers for Disease Control and Prevention, Mailstop D18, 1600 Clifton Rd., Atlanta, GA 30333, USA; fax: 404-639-4504; e-mail: rlm8@cdc.gov.

possible trend, and compare the prevalence with that of local hospitals. Findings were used to guide local treatment and prevention measures.

### The Study

### Prospective Laboratory Survey

To confirm the accuracy of the preliminary antibiotic susceptibility results from Hospital A, we prospectively collected all ***S. pneumoniae*** isolates identified by Hospital A's microbiology laboratory from October 23, 1997, through February 19, 1998. An invasive isolate was defined as any positive culture for ***S. pneumoniae*** obtained from blood; cerebrospinal fluid; joint, pleural, or peritoneal fluid; or other normally sterile site. We confirmed the antibiotic susceptibility profiles of each of these isolates by retesting them at another community facility, Hospital B. To determine antibiotic susceptibility, both hospitals used oxacillin disk screening followed by the antimicrobial gradient disk (E-test). The penicillin susceptibility of the invasive isolates was also confirmed using broth dilution at the laboratory of Children's Hospital of Oklahoma, which serves as the regional pediatric referral hospital in Oklahoma.

For this report, the term ***penicillin nonsusceptible*** (PNSP)is used to describe any ***S. pneumoniae*** organism with reduced susceptibility to penicillin. We used the National Committee for Clinical Laboratory Standards' cut points to identify antibiotic susceptibility and determine whether nonsusceptible organisms had intermediate or high resistance (for penicillin, < 0.06 µg/mL was considered susceptible; 0.10-1.00 µg/mL was intermediate, and > 2.00 µg/mL was resistant) (11).

Hospitals A and B used the E-test to evaluate the susceptibility of each isolate to third-generation cephalosporins, but Hospital A used cefotaxime as the test antibiotic whereas Hospital B used ceftriaxone. Both hospitals also tested isolates for susceptibility to trimethoprim sulfamethoxazole (TMP-S).

The serotypes of 16 of the ***S. pneumoniae*** isolates (the first 13 invasive isolates collected and 3 additional noninvasive isolates) from Hospital A were determined by the Division of Bacterial and Mycotic Diseases Laboratory, Centers for Disease Control and Prevention (CDC). The susceptibility profiles and serotypes of these isolates were compared with those obtained from a 1996 nursing home outbreak of invasive multidrug-resistant ***S. pneumoniae*** in a geographically distant region of Oklahoma (12).

### Hospital A Retrospective Cohort Study

To describe the epidemiology of ***S. pneumoniae*** at Hospital A during 1994 through 1997, we retrospectively identified all patients with invasive isolates by reviewing records of the hospital's microbiology laboratory. After linking these with patient medical records, we extracted information on demographics, medical history, clinical course, and results of antibiotic susceptibility testing of the isolates for each patient. Trends over time were determined by the chi-square for trend test, and potential predictor factors for acquiring nonsusceptible organisms—such as hospitalization within the past year at Hospital A, nursing home residence, and various demographic factors—were evaluated (Epi-Info version 6.04b, CDC).

### 1998 Oklahoma Sentinel Hospital Surveillance

During this investigation, a sentinel hospital surveillance system was started for invasive ***S. pneumoniae*** in Oklahoma. We compared the prevalence of PNSP at Hospital A with data from the 26 sentinel hospitals that participated in this surveillance system in 1998. Ten were among the acute-care hospitals studied in central Oklahoma during 1989 to 1990 (10); the others were scattered throughout the state. Some hospitals used bacterial broth dilution or antimicrobial gradient strips for penicillin susceptibility testing; others used bacterial disk diffusion or an antimicrobial panel. The sentinel hospitals accounted for approximately 58% of all medical and surgical hospital beds in the state.

### Results

### Prospective Laboratory Survey

From October 23, 1997, through February 19, 1998, Hospital A's microbiology laboratory identified ***S. pneumoniae*** isolates from 48 patients. Of these, 17 (35%) were invasive: 2 were from cerebrospinal fluid, and 15 were from blood. The remaining 31 (65%) were noninvasive; 22 isolates were from sputum and 9 from nose, sinus, tonsils, or bronchial washings. The median patient age was 60 years; only 5 (10%) were <18 years of age. Of patients, 26 (54%) were male. Twenty (80%) of the 25 for whom race was known were white, 4 (16%) were black, and l (4%) was Asian. Twenty-nine (60%) of the patients resided in Oklahoma County; the rest were from seven other Oklahoma counties. The isolates were tested at hospitals A and B for susceptibility to several different antibiotics.

Hospital A reported that 31 (65%) of the 48 ***S. pneumoniae*** isolates were not susceptible to penicillin; 8 (17%)had intermediate resistance and 23 (48%) had high penicillin resistance. Twenty-three (48%) isolates were nonsusceptible to cefotaxime, and 13 (27%) were highly cefotaxime resistant. Of 47 isolates tested, 29 (62%) were nonsusceptible to TMP-S; 23 (49%) were highly resistant (Table 1). All isolates were susceptible to vancomycin.

When these isolates were tested for penicillin susceptibility at Hospital B, a similar prevalence of PNSP was reported (62%), though fewer were highly resistant (29%). However, when testing for susceptibility to a third-generation cephalosporin was performed at Hospital B, only 11 (23%) of 48 isolates were reported as nonsusceptible to ceftriaxone, compared with the 23 (48%) nonsusceptible to cefotaxime at Hospital B (Table 1). For all 15 isolates for which cefotaxime susceptibility results at Hospital A differed from ceftriaxone susceptibility results at Hospital B, the difference was in the direction of higher resistance to cefotaxime than to ceftriaxone (sign test, p <0.001).

When penicillin susceptibilities of the 17 invasive isolates tested with the E-test by both hospitals were compared with the susceptibilities determined at Children's Hospital of Oklahoma using broth dilution, the results were again similar. For 11 (65%) of the invasive isolates, all three test results were interpreted as the same. When discordance was noted, the differences were minor (i.e., susceptible was interpreted as intermediate [or vice versa], or intermediate was interpreted as resistant [or vice versa]). No major differences occurred in interpretation of penicillin susceptibility (susceptible to resistant or resistant to susceptible) (Table 2).

Among the 16 serotyped isolates, 10 serotypes were identified: 4, 6A, 9V (3 isolates), 12F, 18C, 19A (2 isolates), 22F, 23F (4 isolates), 33F, and 35B. Only two (12.5%)(6A and 35B) would not have been covered by pneumococcal vaccine. Among the 23F isolates, two were invasive, and two were not. One of the invasive

Table 1. Antibiotic-susceptibility test results[a] for 48 isolates of ***Streptococcus pneumoniae*** from Hospital A, Oklahoma, October 23, 1997, through February 19, 1998, and retest results from Hospital B

| | Hospital A No. (%) | Hospital B No. (%) |
|---|---|---|
| Oxacillin disk | | |
| Sensitive | 16/47 (34) | 17/46 (37) |
| Resistant | 31/47 (66) | 29/46 (63) |
| Penicillin | | |
| Sensitive | 17/48 (35) | 18/48 (38) |
| Intermediate | 8/48 (17) | 16/48 (33) |
| Resistant | 23/48 (48) | 14/48 (29) |
| Ceftriaxone | | |
| Sensitive | not performed | 37/48 (77) |
| Intermediate | | 7/48 (15) |
| Resistant | | 4/48 (8) |
| Cefotaxime | | |
| Sensitive | 25/48 (52) | not performed |
| Intermediate | 10/48 (21) | |
| Resistant | 13/48 (27) | |
| Trimethoprim-sulfamethoxazole | | |
| Sensitive | 18/47 (38) | 16/47 (34) |
| Intermediate | 6/47 (13) | 2/47 (4) |
| Resistant | 23/47 (49) | 29/47 (62) |

[a]By E-test and oxacillin disc.

Table 2. Penicillin-susceptibility test results from three laboratories for 17 invasive isolates of ***Streptococcus pneumoniae***, Hospital A, Oklahoma, November 1997 through February 1998

| | E-test | | Broth dilution |
|---|---|---|---|
| Source | Hospital A | Hospital B | Children's Hospital of Oklahoma |
| Blood | I | S | S |
| Blood | R | I | R |
| Blood | R | R | R |
| Blood | R | I | I |
| Blood | S | S | S |
| Blood | I | I | I |
| Blood | R | R | I |
| Blood | S | S | S |
| Blood | R | I | I |
| Blood | S | S | S |
| CSF | S | S | S |
| Blood | S | S | S |
| Blood | I | I | I |
| CSF | R | R | I |
| Blood | R | R | R |
| Blood | R | R | R |
| Blood | S | S | S |
| I or R % | 65% | 59% | 59% |

I, having intermediate resistance; R, highly resistant; S, susceptible to the antibiotic.
CSF = cerebrospinal fluid

23F isolates was highly sensitive to everything tested; the other three were highly resistant to several antibiotics and had similar susceptibility profiles to the 23F isolates identified in the nursing home outbreak months earlier (12).

### Hospital A Retrospective Cohort Study

Review of the epidemiology of ***S. pneumoniae*** infection at Hospital A during 1994 through 1997 revealed 71 case-patients with invasive infections. Forty-three (61%) were female, 62 (87%) were white, and 7 (10%) were black. The median age was 64 (range: 1 to 94), and 21 (30%) patients died. Twenty-three (32%) had been hospitalized at Hospital A during the previous 6 months. Nineteen (27%) were residents of a nursing home, 51 (72%) acquired infection in the community, and one (1%) acquired infection while hospitalized for another illness. Five (7%) had asplenia. Sixty-five (92%) isolates were from blood; four (6%) from the joint, pleural, or peritoneal fluid; and one (1%) each from cerebrospinal fluid and an aortic graft. Fifty-six (79%) infected patients resided in Oklahoma City or its vicinity.

Fifty-eight (82%) patients with invasive ***S. pneumoniae*** infections during this period fit the Advisory Committee on Immunization Practices' (ACIP) criteria for pneumococcal vaccine (13). Of the 21 deaths, 20 (95%) would have met the criteria. Only one patient's record, however, showed receipt of pneumococcal vaccine. This patient had had a splenectomy and did not survive the infection.

For the 71 case-patients, 27 (38%) isolates were PNSP, and 13 (18%) were highly resistant. Fifteen (21%) were nonsusceptible to cefotaxime, and one (1%) was highly resistant. All isolates tested were sensitive to vancomycin and clindamycin. Forty-two (59%) patients with invasive pneumococcal disease initially received ceftriaxone, and 3 (4%) received vancomycin; 20 (28%) received vancomycin at some point during their hospital stay. Case-patients with PNSP infections were not more likely to die than patients with penicillin-susceptible infections.

Over the 4-year period of the study, a trend toward increased nonsusceptibility to penicillin was noted at Hospital A (1 of 9, 8 of 24, 7 of 20, 11 of 18; $p = 0.02$). The same trend was noted for cefotaxime nonsusceptibility (0 of 9, 3 of 24, 5 of 20, 9 of 18; $p = 0.008$). A significant trend toward increased penicillin nonsusceptibility was noted in nursing home patients (0 of 2, 2 of 6, 4 of 7, 4 of 4; $p < 0.05$) but not in non-nursing home residents (2 of 7, 7 of 18, 4 of 13, 7 of 14; $p > 0.05$). Nursing home residents were more likely than non-nursing home residents to have a nonsusceptible strain of ***S. pneumoniae,*** but this finding was not significant (10 of 18 vs. 20 of 53, risk ratio [RR] = 1.5; 95% confidence interval [CI] = 0.9 – 2.5; $p > 0.05$). Similarly, patients who had been hospitalized at Hospital A in the previous 6 months were more likely to have PNSP infections than those who had not been hospitalized there, but again the association was not significant (18 of 33 vs. 12 of 38, RR = 1.7; 95% CI = 1.0-3.0; $p = 0.09$).

### 1998 Oklahoma Sentinel Surveillance

Surveillance data were available from 26 sentinel hospital laboratories in Oklahoma during 1998, including Hospital A's. Seventeen laboratories used the E-test, four used broth dilution, three used disk diffusion (Kirby Bauer), and two used an antimicrobial panel (Microscan) for susceptibility testing. Of 282 invasive isolates tested, 197 (70%) were penicillin susceptible; 52 (18%) had intermediate resistance, and 33 (12%) were highly penicillin resistant. Of the 26 sentinel hospitals, 13 had at least 10 invasive isolates of ***S. pneumoniae*** during 1998. The prevalence of PNSP ranged from 10% to 45% in these 13 hospitals.

### Discussion

The high prevalence of PNSP invasive isolates observed among vaccine-eligible, elderly adults from Hospital A in Oklahoma City is similar to that recently reported as the highest of a range of proportions from CDC's Emerging Infections Program's Active Bacterial Core Surveillance for 1997 (8). Penicillin nonsusceptibility varied among Oklahoma sentinel hospitals, but overall it has increased markedly since studies were conducted in Oklahoma in the 1980s (9,10).

The increase in penicillin nonsusceptibility at Hospital A appears to be the result of a trend over the past 4 years. While we found no evidence of an increase in the number of patients with invasive ***S. pneumoniae*** coming from nursing homes, we did note an increasing prevalence of PNSP among such patients.

The community in which Hospital A is located is fairly affluent. Resistance levels can vary within a city, and more affluent or suburban communities may have higher rates of antibiotic resistance among ***S. pneumoniae*** isolates (5), perhaps attributable to more frequent use of antibiotics. Some resistant isolates were similar to those detected in a recent nursing home outbreak involving a single clone of a highly resistant organism (12). Contribution of an outbreak clone to the high prevalence observed here is possible; however, our evidence suggests that several resistant serotypes contributed to the observed increase. Although the reason for the PNSP increase at Hospital A is unclear, local antibiotic prescribing practices and one or more highly resistant clones circulating in the community or nursing homes may have been involved.

This investigation had at least three limitations. First, the number of invasive isolates for the retrospective study was small, and specific risk factors for acquiring PNSP at Hospital A were not identified. Thus, no single factor explains the high rates observed. Other studies have suggested that age (< 6, or > 65), race, recent antibiotic use, socioeconomic status, and geographic factors may be associated with resistance (2,5). Second, because of the small number of isolates collected prospectively beginning in October 1997, we could not confirm or explain the significant difference in third-generation cephalosporin susceptibility reported by hospitals A and B. Although these facilities used two different test antibiotics (cefotaxime and ceftriaxone) to determine susceptibility, the genetic mechanism responsible for cephalosporin resistance would have been expected to have made the same organisms equally susceptible to either antibiotic. If confirmed, this finding is relevant in clinical settings where cefotaxime is used to test for susceptibility and ceftriaxone is used for treatment because ***S. pneumoniae*** may not have equal susceptibility to both antibiotics. Although we found reliability among the three laboratories for determining penicillin susceptibility, consistent with reports in the microbiology literature (14,15), the lack of interlaboratory reliability in differentiating between high and intermediate penicillin resistance was also unexplained. Finally, we did not assess whether treatment failure contributed to illness and death. Although case-patients with PNSP infections were not more likely to die than patients with penicillin-susceptible infections, the impact of underlying illnesses and the virulence of the infecting strain on the outcome is not known.

For Hospital A and the state of Oklahoma, our findings have implications for disease treatment and prevention. Surveillance, increased use of the pneumococcal vaccine, and judicious use of antimicrobial drugs are important components of the effort to limit the spread of resistant ***S. pneumoniae*** (3,13,16). For determining community-specific prevalence of PNSP infections, use of hospital antibiograms has been shown to be comparable to active surveillance with centralized testing (17). However, our investigation suggested that when different third-generation cephalosporin test antibiotics are used and when determining high versus intermediate penicillin nonsusceptibility, significant differences can occur between hospital laboratories in the determination of susceptibility. Such differences, whatever their explanation, have important clinical implications for the optimal use of antibiotics (e.g., vancomycin, ceftriaxone, and fluoroquinolones). Accurate laboratory information is needed at the local level to optimize use of antibiotics and minimize the development of antibiotic resistance. Accurate surveillance information is needed at the state level to compare regional prevalences. Further study with more isolates is needed to identify ways to improve the accuracy of this surveillance system.

This investigation documented that many cases of invasive pneumococcal disease could have been prevented by improved immunization practices: 82% of Hospital A case-patients during 1994 to 1997 were eligible for vaccine by ACIP criteria, but only one had record of receiving it. Although hospital records may not have reflected true vaccination status, 1997 data from the Behavioral Risk Factor Surveillance System showed that 40% of Oklahoma residents > 65 reported having received the pneumonia vaccine, which is less than the national goal of >60% (18). Targeting vaccination programs at nursing homes may be particularly effective. Oklahoma has recently required that all nursing homes offer pneumococcal vaccination to their residents and provide documentation of vaccination status.

In addition, Hospital A instituted a hospital-based pneumococcal immunization program with standing orders to vaccinate inpatients aged 65 years who are eligible for the vaccine based on ACIP criteria (13). Because nearly one out of three of the patients with invasive pneumococcal disease admitted to Hospital A during 1994 to 1997 had been hospitalized there in the previous 6 months, an inpatient screening and vaccination program could have prevented a substantial number of cases. Hospital-based programs have been shown to be effective in vaccinating high-risk adults in other settings (19-21), and the use of standing orders to vaccinate eligible adults with pneumococcal vaccine has been recommended by the Task Force on Community Preventive Services (22). Adult vaccination programs in nontraditional settings (e.g., pharmacies, churches, and the workplace) might further raise vaccination coverage in this area (23).

Finally, efforts to promote judicious use of antibiotics are needed to minimize the spread of antibiotic-resistant ***S. pneumoniae***. Several studies have demonstrated that antimicrobial drug use is associated with resistance in ***S. pneumoniae*** (24-26). A combination of interventions involving education of both physicians and patients has been successful in reducing antibiotic use (27) and would likely reduce the trend of increasing resistance in Oklahoma.

### Acknowledgments

The authors thank June Ketchum, Dennis Zinn, Everett Dodd, Keeta Gilmore, Denise Robison, and Carolyn Bradley for susceptibility testing of isolates; the surveillance coordinators at the 26 sentinel hospitals for providing data on invasive ***S. pneumoniae;*** John Elliott and Jay Butler for assistance with serotyping isolates and interpreting the results; and Andy Pelletier and Cyndy Whitney for insightful comments.

Dr. Moolenaar is a CDC medical epidemiologist. He was serving as Deputy State Epidemiologist in Oklahoma at the time of this study.

### References

1. Breiman RF, Butler JC, Tenover FC, Elliott JA, Facklam RR. Emergence of drug-resistant pneumococcal infections in the United States. JAMA 1994;271:1831-5.
2. Butler JC, Hoffman J, Cetron MS, Elliott JA, Facklam RR, Breiman RF, et al. The continued emergence of drug-resistant ***Streptococcus pneumoniae*** in the United States: an update from the Centers for Disease Control and Prevention's pneumococcal sentinel surveillance system. J Infect Dis 1996;174:986-93.
3. Centers for Disease Control and Prevention. Defining the public health impact of drug-resistant ***Streptococcus pneumoniae***: report of a working group. MMWR Morb Mortal Wkly Rep 1996;45(RR-1):1-20.
4. Pastor P, Medley F, Murphy TV. Invasive pneumococcal disease in Dallas County, Texas: results from population-based surveillance in 1995. Clin Infect Dis 1998;26:590-5.
5. Hoffman J, Cetron MS, Farley MM, Baughman WS, Facklam RR, Elliott JA, et al. The prevalence of drug-resistant ***Streptococcus pneumoniae*** in Atlanta. N Engl J Med 1995;333:481-6.
6. Heffernan R, Henning K, Labowitz A, Hjelte A, Layton M. Laboratory survey of drug-resistant ***Streptococcus pneumoniae*** in New York City, 1993-1995. Emerg Infect Dis 1998;4:113-6.
7. Butler JC, Cetron MS. Pneumococcal drug resistance: the new "special enemy of old age." Clin Infect Dis 1999;28:730-5.
8. Centers for Disease Control and Prevention. Geographic variation in penicillin resistance in ***Streptococcus pneumoniae***—selected sites, United States, 1997. MMWR Morb Mortal Wkly Rep 1999:48;656-61.
9. Istre GR, Tarpay M, Anderson M, Pryor A, Welch D, the Pneumococcus Study Group. Invasive disease due to ***Streptococcus pneumoniae*** in an area with a high rate of relative penicillin resistance. J Infect Dis 1987;156:732-5.
10. Haglund LA, Istre GR, Pickett DA, Welch DF, Fine DP, and the Pneumococcus Study Group. Invasive pneumococcal disease in central Oklahoma: emergence of high-level penicillin resistance and multiple antibiotic resistance. J Infect Dis 1993;168:1532-6.
11. National Committee for Clinical Laboratory Standards. Performance standards for antimicrobial susceptibility testing (5th informational supplement). NCCLS document no. M100-S5. Villanova, PA: The Committee, 1994.
12. Nuorti JP, Butler JC, Crutcher JM, Guevara R, Welch D, Holder I, et al. An outbreak of multidrug-resistant pneumococcal pneumonia and bacteremia among unvaccinated nursing home residents. N Engl J Med 1998;338:1861-8.
13. Centers for Disease Control and Prevention. Prevention of pneumococcal disease: recommendations of the Advisory Committee on Immunization Practices (ACIP). MMWR Morb Mortal Wkly Rep 1997:46(RR-8):1-24.
14. Macias EA, Mason EO Jr, Ocera HY, La Rocco MT. Comparison of E test with standard broth microdilution for determining antibiotic susceptibilities of penicillin-resistant strains of ***Streptococcus pneumoniae***. J Clin Microbiol 1994;32:430-2.
15. Tenover FC, Baker CN, Swenson JM. Evaluation of commercial methods for determining antimicrobial susceptibility of ***Streptococcus pneumoniae***. J Clin Microbiol 1996;34:10-4.
16. Jernigan DB, Cetron MS, Breiman RF. Minimizing the impact of drug-resistant ***Streptococcus pneumoniae*** (DRSP): a strategy from the DRSP Working Group. JAMA 1996;275:206-9.
17. Chin AE, Hedberg K, Cieslak PR, Cassidy M, Stefonek KR, Fleming DB. Tracking drug-resistant ***Streptococcus pneumoniae*** in Oregon: an alternative surveillance method. Emerg Infect Dis 1999;5:688-93.
18. Centers for Disease Control and Prevention. Influenza and pneumococcal vaccination levels among adults aged greater than or equal to 65 years—United States. MMWR Morb Mortal Wkly Rep 1998:47;797-802.

19. Klein RS, Adachi N. An effective hospital-based pneumococcal immunization program. Arch Intern Med 1986;146:327-9.
20. Vondracek TG, Pham TP, Huycke MM. A hospital-based pharmacy intervention program for pneumococcal vaccination. Arch Intern Med 1998;158:1543-7.
21. Gyorkos TW, Tannenbaum TN, Abrahamowicz M. Evaluation of the effectiveness of immunization delivery methods. Can J Public Health 1994;85(Suppl):S14-30.
22. Centers for Disease Control and Prevention. Vaccine-preventable diseases: improving vaccination coverage in children, adolescents and adults. A report on recommendations of the Task Force on Community Preventive Services. MMWR Morb Mortal Wkly Rep 1999;48(RR-8):1-15.
23. Centers for Disease Control and Prevention. Adult immunization programs in nontraditional settings: quality standards and guidance for program evaluation—a report of the National Vaccine Advisory Committee and use of standing orders programs to increase adult vaccination rates: recommendations of the Advisory Committee on Immunization Practices. MMWR Morb Mortal Wkly Rep 2000;49(RR-1):1-13.
24. Bedos JP, Chevret S, Chastang C, Geslin P, Regnier B, and the French Cooperative Pneumococcus Study Group. Epidemiological features of and risk factors for infection by ***Streptococcus pneumoniae*** strains with diminished susceptibility to penicillin: findings of a French survey. Clin Infect Dis 1996;22:63-72.
25. Nava JM, Bella F, Garau J, Lite J, Morera MA, Marti C, et al. Predictive factors for invasive disease due to penicillin-resistant ***Streptococcus pneumoniae***: a population based study. Clin Infect Dis 1994;19:884-90.
26. Frick PA, Black DJ, Duchin JS, Delaganis S, Mckee WM, Fritsche TR. Prevalence of antimicrobial drug-resistant ***Streptococcus pneumoniae*** in Washington state. West J Med 1998;169:364-9.
27. Gonzales R, Steiner JF, Lum A, Barret PH. Decreasing antibiotic use in ambulatory practice: impact of a multidimensional intervention on the treatment of uncomplicated acute bronchitis in adults. JAMA 1999;281:1512-9.
